# Dietary supplements for aggressive behaviour in people with intellectual disabilities: A randomised controlled crossover trial

**DOI:** 10.1111/jar.13041

**Published:** 2022-10-12

**Authors:** David A. A. Gast, Robert Didden, Johanna J. Westera, Ondine van de Rest, Albert M. van Hemert, Erik J. Giltay

**Affiliations:** ^1^ Department of Psychiatry Leiden University Medical Center Leiden The Netherlands; ^2^ Gemiva‐SVG Group Gouda The Netherlands; ^3^ Behavioural Science Institute Radboud University Nijmegen The Netherlands; ^4^ Trajectum Zwolle The Netherlands; ^5^ 's Heeren Loo Amersfoort The Netherlands; ^6^ Division of Human Nutrition and Health Wageningen University & Research Wageningen The Netherlands

**Keywords:** aggressive behaviour, dietary supplements, intellectual disabilities, randomised controlled trial

## Abstract

**Background:**

Aggressive incidents are common in people with intellectual disabilities. Therefore, we aimed to assess whether supplementation of multivitamins, minerals, and omega‐3 fatty acids (FA) reduces aggressive incidents.

**Methods:**

We conducted a randomised, triple blind, placebo controlled, single crossover intervention trial. People with intellectual disabilities or borderline intellectual functioning, between 12 and 40 years of age, and showing aggressive behaviour were included. Participants received either a daily dose of dietary supplements, or placebo. Primary outcome was the number of aggressive incidents, measured using the Modified Overt Aggression Scale (MOAS).

**Results:**

there were 113 participants (placebo, *n* = 56), of whom 24 (placebo, *n* = 10) participated in the crossover phase of the trial. All 137 trajectories were included in the analyses. There was no significant difference in mean number of aggressive incidents per day between those assigned to supplements and those who received placebo (rate ratio = 0.93: 95% Confidence Interval [CI] = 0.59–1.45).

**Conclusion:**

In this pragmatic trial, we did not find significant differences in the outcomes between the supplement and placebo arms. The COVID‐19 pandemic started midway through our trial, this may have affected the results.

## 1 INTRODUCTION

Aggressive behaviour is common in people with intellectual disabilities. Prevalence rates range from 10% to more than 45% depending on the definitions of aggressive behaviour, the sub‐population studied and the measurement methods used (Bowring et al., 2019; Didden et al., 2016; Drieschner et al., 2013). Much can be done to reduce aggression, for example through the use of anger management interventions, behavioural therapies, contextual approaches, sedatives, and off‐label antipsychotics (Didden et al., 2019; Lloyd & Kennedy, 2014). However, other evidence‐based and safe treatment options remain necessary (Didden et al., 2016; Scheifes, 2015).

In vivo and in vitro research has revealed multiple mechanisms of action by which micronutrients may influence the central nervous system (CNS), including neurotransmitter synthesis, energy production and neuroprotective properties (Calderon‐Ospina & Nava‐Mesa, 2020; Kennedy, 2016; Khanna et al., 2006; Parletta et al., 2013). A sub‐optimal functioning CNS is associated with reduced self‐control and aggressive behaviour (Jackson, 2016). There also is accumulating evidence for the hypothesis that dietary supplements may reduce aggressive behaviour (Benton, 2007; Frensham et al., 2012; Rucklidge & Kaplan, 2013). A decrease in antisocial behaviour was found in four randomised trials for multivitamins and minerals on inmates' behaviour (Gesch et al., 2002; Schoenthaler et al., 1997; Schoenthaler et al., 2021; Zaalberg et al., 2010). Positive effects of dietary supplements on externalising behaviour in children with and without mental health problems were found in another four randomised trials (Adams et al., 2011; Raine et al., 2016; Rucklidge et al., 2018; Schoenthaler & Bier, 2000). Although there are also two randomised trials in students that showed inconclusive results (Long & Benton, 2013; Tammam et al., 2016), the overall effect of the supplements versus placebo on antisocial behaviour was statistically significant and in favour of the active supplements (Benton, 2007; Rucklidge & Kaplan, 2013).

Given the effectiveness in randomised controlled trails (RCTs) among other study populations, we conducted a randomised trial on the effectiveness of dietary supplements to reduce aggressive incidents in people with intellectual disabilities. Other studies on aggressive behaviour in people with intellectual disabilities have shown that recruiting enough participants can be a problem (Oliver‐Africano et al., 2010). In order to achieve sufficient statistical power with a relative small number of participants (Richens, 2001), we added a crossover arm after the second year of recruitment. We found little information about the carry over effect of the combination of dietary supplements used, so we chose to use the same wash out time for participating in the crossover part as for the initial inclusion. Our hypothesis was that the supplementation of vitamins, minerals and omega‐3 FA would lead to a reduction in aggressive behaviour in people with intellectual disabilities and borderline intellectual functioning. Our second hypothesis was that this intervention would also improve their quality of life.

## 2 MATERIAL AND METHODS

### 2.1 Design and procedure

This study was a pragmatic, randomised, triple‐blind, placebo controlled, multicentre and crossover intervention study to investigate the effect of dietary supplements on aggressive behaviour among people with intellectual disabilities and borderline intellectual functioning. The study was registered at the Clinical Trials Register (NCT03212092). Approval for conducting the study was granted by the Medical Ethics Committee of the Leiden University Medical Center (LUMC) (NL60839.058.17). The coordinating centre was the LUMC department of psychiatry, and the data was collected at six care organisations between 11 April 2018 and 1 February 2021. Participants first entered a run‐in phase and received placebo supplements for 2 weeks. Thereafter, they were randomised and included in the 16‐week study. After completion, they were asked to participate in the crossover trial, and after a new informed consent procedure and a washout period of at least 2 weeks, they would repeat the study in a different treatment arm, while the study pharmacist maintained the blind to treatment allocation. Support staff offered the supplements and reported incidents daily. Trained research assistants collected baseline and endpoint data from the support staff, and if possible, from the participants. On a weekly basis, they monitored incident reports and adverse events collected by support staff. The participants received a gift voucher of 5 euros twice for their contribution to providing baseline and endpoint data.

### 2.2 Participants

Participants were recruited from six care organisations for people with intellectual disabilities in different regions in the Netherlands (i.e., Amarant, Amerpoort, Gemiva‐SVG‐groep, Schakenbosch, 's Heeren Loo and Trajectum). People with borderline intellectual functioning may need similar support as people with mild intellectual disability due to psychological co‐morbidity and deficits in adaptive abilities (Jonker et al., 2021). In the Netherlands they can receive support through the care system for people with intellectual disabilities. Therefore, these people were also recruited to participate in our study. To explain the study to potential participants an animation film and folders in simple language were developed. People who were willing to participate were asked to provide informed consent. For legally incapacitated people with intellectual disabilities, as monitored by the organisations' psychologist, and children under the age of 16, informed consent was (also) requested from the legal representative.

The following inclusion and exclusion criteria were used: Successfully completing the run‐in phase; Age between 12 and 40 years; Receiving care from an intellectual disabilities‐organisation; IQ < 85; Score ≥5 on the Social Dysfunction and Aggression Scale (see Measurements); Not pregnant or breast feeding; Does not have one of the following conditions: Williams syndrome, Wilson's diseases, hemochromatosis or hyperparathyroidism; Not using levothyroxine, methyldopa or levodopa; No fish allergy; Not using dietary supplements with vitamins or minerals for the past 14 days (only vitamin D supplements up to 50 μg per day were allowed).

### 2.3 Intervention

Participants received four capsules daily with one meal, consisting of 2× multivitamin minerals and 2x omega‐3 FA. The multivitamin minerals contained 12 vitamins and 9 minerals and consisted of a powdered multivitamin tablet (Bonusan Multi Vital Actief) divided into two opaque, size ‘0’ capsules. The omega‐3 supplements (Bonusan Omega‐3 Forte) contained 200 mg DHA and 300 mg EPA and were bovine gelatin soft gel capsules with an opaque coating. As can be seen in Appendix 1, Supporting information, the daily dose of the micronutrients used in our study is in the range of doses used in other studies on the effect of dietary supplements on behaviour. The placebos were visually indistinguishable from the active supplements according to a test panel of staff workers and people with mild intellectual disabilities and borderline intellectual functioning. A vanilla scented silica gel sachet was added to each jar of supplements and placebos to give them a similar scent. For the placebo capsule contained a small amount (0.8 mg) of riboflavin. The supplements/placebos were administered by the LUMC research pharmacy and was ordered by the researcher using a unique randomly assigned participant code.

### 2.4 Randomization

Block randomization was used with a block size of 8 participants at a 1: 1 ratio through a computerised random number generator. Four strata were made according to age (i.e., younger than 18 or 18 and older) and aggression score in the preceding week (low aggression [SDAS <18] or high aggression [SDAS ≥18]). The allocation was managed by an independent LUMC research pharmacist and only released upon completion of the statistical analysis on the primary outcome.

### 2.5 Measurements

The primary outcome was the sum of the aggressive incidents at either the residential or the daycare facility, as reported daily with the modified overt aggression scale (MOAS) by the support staff (Kay et al., 1988; Silver & Yudofsky, 1991; Sorgi et al., 1991). The MOAS is a reliable tool to measure aggressive behaviour in people with intellectual disabilities (Cohen et al., 2010), and has an intraclass correlation coefficient (ICC) of 0.93 (Oliver et al., 2007). Four types of aggression are reported using this scale: verbal, against objects, physical, and self‐harm. The severity of the incidents were scored on a scale from 0 (i.e., mild) to 4 (i.e., extreme) for each type of aggressive behaviour. On the MOAS we added a daily record of whether the supplements had been taken. The MOAS was completed daily by the support staff and monitored weekly for clarity and completeness by the research assistants. If the data was incorrect or missing, the assistant would call the support staff for clarification. The support staff of all participating sites were trained on site to report the aggressive incidents using the MOAS.

As a secondary outcome, quality of life was measured with the Intellectual Disability Quality of Life Scale (IDQOL‐16). This self‐report scale consists of 16 statements, which were visualised with pictograms and were scored on a 5‐point Likert scale in the shape of faces (smiley's), with the leftmost face smiling and the rightmost face looking angry (Hoekman et al., 2001). The score ranges from 16 to 80, with higher scores indicating a better QoL. The Cronbach's alpha in our sample was 0.87. The IDQOL‐16 was completed at baseline and in the last week of the trial. If the participant was unable to complete the scale, the support staff was asked to help complete it as a proxy.

The social dysfunction and aggression scale (SDAS‐11) is an 11‐item observer‐rated questionnaire used to measure social dysfunction and aggressive behaviour during the previous week. Support staff scored each item on a 5‐point Likert scale, ranging from 0 (not present) to 4 (extremely severe). The total score ranges from 0 to 44, with higher scores indicating more social dysfunctional and aggressive behaviour (Wistedt et al., 1990). Psychometric qualities of the SDAS were found to be acceptable to good (Kobes et al., 2012). Cronbach's alpha in our sample was 0.88. The SDAS‐11 was completed by a support staff of the participant at baseline and in the last week of the trial.

The Dutch Healthy Diet Food Frequency Questionnaire (DHD) can be used to estimate the extent to which the eating pattern is in accordance with the Dutch guidelines for a healthy diet from 2015 (Looman et al., 2017). It has 40 items and yields a DHD index score ranging from 0 to 160, with higher scores indicating a better diet quality. The scale is made up of 16 components, namely: vegetables, fruits, whole wheat products, legumes, nuts, dairy, fish, tea, fats and oils, coffee, red meats, processed meats, sugar containing beverages, alcohol, salt and unhealthy food products. The scale has acceptable concurrent validity and can be used for epidemiological studies (van Lee et al., 2016). The DHD was completed at baseline by the support staff and participant (if possible).

We used case file data provided by the healthcare organisations to obtain IQ scores, medication, autism spectrum diagnosis, and demographic characteristics of participants. This information was collected at baseline by the research assistant.

### 2.6 Sample size

The primary outcome measure was the number of aggressive incidents measured with the MOAS. The power calculation was based on an effect size of incidence rate ratio (IRR) = 0.75 with an *α* of .05 in order to achieve a power of at least .80. This is a low estimate derived from the effect sizes found in previous RCTs (Gesch et al., 2002; Zaalberg et al., 2010). This yielded a sample size of at least 126, with at least 18 crossover participants.

### 2.7 Statistical analyses

Characteristics and outcomes were summarised as means with standard deviations (SD) for continuous variables, and as numbers and proportions for categorical variables. Adherence proportion was calculated by dividing the number of days the supplements were offered by the number of days the supplements were taken. The MOAS data was calculated in two ways. First, we summed all counts (number of marks). In addition, the sum of the counts per any of the four types of aggression was calculated (i.e., verbal, against objects, physical and self‐harm). Because of the crossover design, we used a generalised linear mixed model (GLMM). The GLMM was preferred over a generalised linear model (GLM) to allow statistical testing based both on both between‐group and within‐subject variance. Those that crossed‐over were added as repeated measurements in the model. The negative binomial distribution was used for the analyses, since the dispersion statistic of the count data was expected to be higher than one (De Bles et al., 2022; Gesch et al., 2002; Zaalberg et al., 2010). The frequency of aggressive incidents was presented as the estimated mean number of incidents per day. The log number of days in the trial was used as offset variable. As a dependent variable, the total number of incidents and four types of incidents were entered consecutively. In order to investigate the trend of the incidence rate ratio (IRR) over time according to the intervention, a negative binomial regression was performed for each period of 10 days separately, of which the estimated means were plotted over time.

For the secondary outcomes, the endpoint minus baseline was calculated and the difference between the active and placebo group was analysed with a linear mixed model analysis. The difference in the number of reported adverse events among the randomised groups was tested with chi‐squared test.

The governmental measures on COVID‐19, such as closing down the daycare centers, social distancing, and restricted visiting of family has had an impact on the incidence rate and types of aggression incidents in people with intellectual disabilities (Gleason et al., 2021; Schuengel et al., 2020). Because COVID‐19 may have affected the outcome of our study, the main analyses were also performed with ‘COVID‐19’ as dichotomous covariate. We used 17 March 2021 as cut‐off point to distinguish trajectories pre and during the pandemic (being the date of the closing down of most daycare centers in the Netherlands). We made three additional analyses to explore the effects of COVID‐19 on our study. First, we entered ‘COVID‐19’ as covariate in the GLMM model, and also calculated the interaction between the intervention and COVID‐19 using a generalised linear model (GLM). Second, we explored the effect of COVID‐19 on aggressive behaviour in our sample, by entering ‘COVID‐19’ as predictor and ‘treatment condition’ as covariate in the GLMM model. Third, we used an independent *t*‐test to test for selective differences in the pre and during COVID‐19 samples for age, body mass index (BMI), diet quality and IQ.

Blinding was tested by asking participants and support staff at the final assessment in the trial whether they thought participants had been taking the active supplements or the placebo. With chi‐squared test we checked whether participants and staff gave the correct answer more often than expected by chance.

Analyses were performed using IBM SPSS statistical software (version 27, IBM Corp Released 2020, IBM SPSS Statistics for Windows), and forest plots and figures using R with RStudio (R version 3.6.0; R Foundation for Statistical Computing, Vienna, Austria, 2016. URL: https://www.R-project.org/).

## 3 RESULTS

**FIGURE 1:**
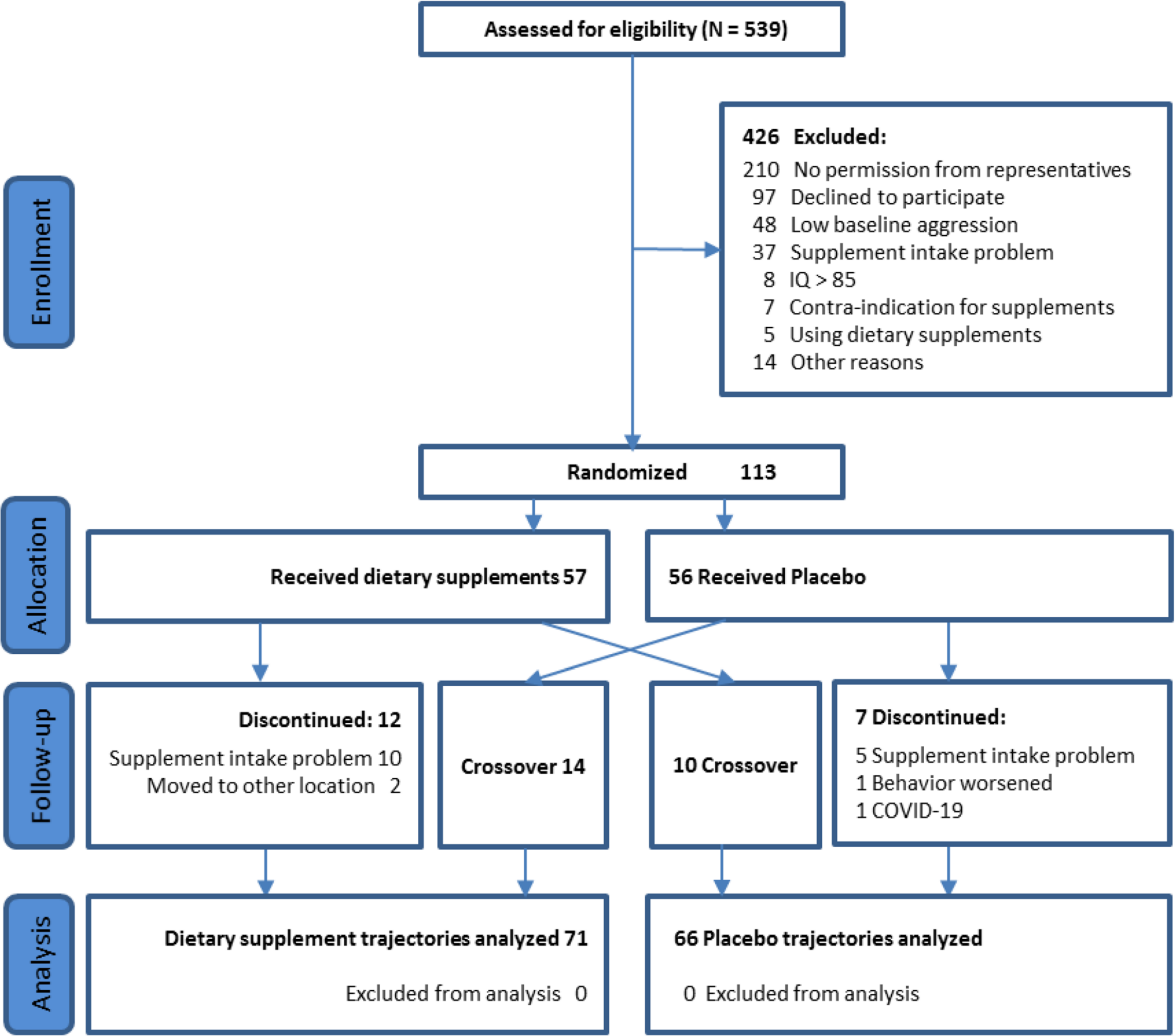
Flowchart of inclusion of participants in the trial [Color figure can be viewed at wileyonlinelibrary.com]

The flowchart of the recruitment is presented in Figure 1. We approached 539 people to participate, 426 were excluded mainly because they did not want to participate or their legal representative did not give consent. Of the 113 participants (57 active, 56 placebo), 24 (14 active, 10 placebo) progressed to the crossover trial, yielding in total 137 treatment trajectories. Socio‐demographic and baseline characteristics of participants are presented in Table 1. Mean age was 22.8 years (SD 7.2), and 34.5% were female. The level of intellectual disabilities varied from profound and severe (*n* = 40), moderate (*n* = 18), to mild ID (*n* = 28) and borderline intellectual functioning (*n* = 27). There were some differences between the initial and the crossover trial, with the participants in the crossover having a higher mean age of 26.3 y (SD = 6.6) versus 22.8 y (SD = 7.2), a higher mean diet quality of 87.9 (SD = 13.0) versus 80.9 (SD = 16.7), and a lower mean IQ of 31.9 (SD = 11.1) versus 48.6 (SD = 20.7). In the participants with a crossover the average time interval between both interventions was 36.1 weeks (*SD* 26.5).

**TABLE 1:** Socio‐demographic and baseline characteristics according to randomised groups

	Active (*n* = 57)	Placebo (*n* = 56)
Demographics		
Age (year)	22.9 (7.1)	22.8 (7.3)
Female gender	22 (38.6%)	17 (30.4%)
Living with parents	4 (7.0%)	5 (8.9%)
BMI	24.2 (4.9)	25.5 (7.0)
Diet quality	79.7 (16.2)	82.0 (17.2)
Smoking	12 (21.1%)	18 (32.1%)
IQ and severity of ID		
IQ	47.2 (19.9)	50.0 (21.6)
Severe to profound ID	21 (36.8%)	19 (33.9%)
Moderate ID	9 (15.8%)	9 (16.1%)
Mild ID	16 (28.1%)	12 (21.4%)
Borderline IF	11 (19.3%)	16 (28.6%)
Clinical data		
SDAS‐11 (baseline)	17.1 (6.3)	17.3 (7.7)
IDQOL‐16 (baseline)	58.6 (9.8)	57.4 (9.2)
Medication and therapy		
Any medication	48 (84.2%)	47 (83.9%)
Antipsychotics	26 (45.6%)	29 (51.8%)
Antiepileptics	4 (7.0%)	6 (10.7%)
Behaviour therapy	4 (7.0%)	10 (17.9%)
Psychiatric co‐morbidity		
Autism	26 (45.6%)	22 (39.3%)
ADHD	5 (8.8%)	6 (10.7%)

*Note*: In brackets is the percentage of the group (%), or the standard deviation (SD) if the value is an outcome score.

Abbreviations: ADHD, attention‐deficit/hyperactivity disorder; BMI, body mass index; ID, intellectual disability; IDQOL‐16, intellectual disability quality of life‐16; IF, intellectual functioning; IQ, intelligence quotient; SDAS‐11, social dysfunction and aggression scale‐11.

### 3.1 Primary outcome

**FIGURE 2:**
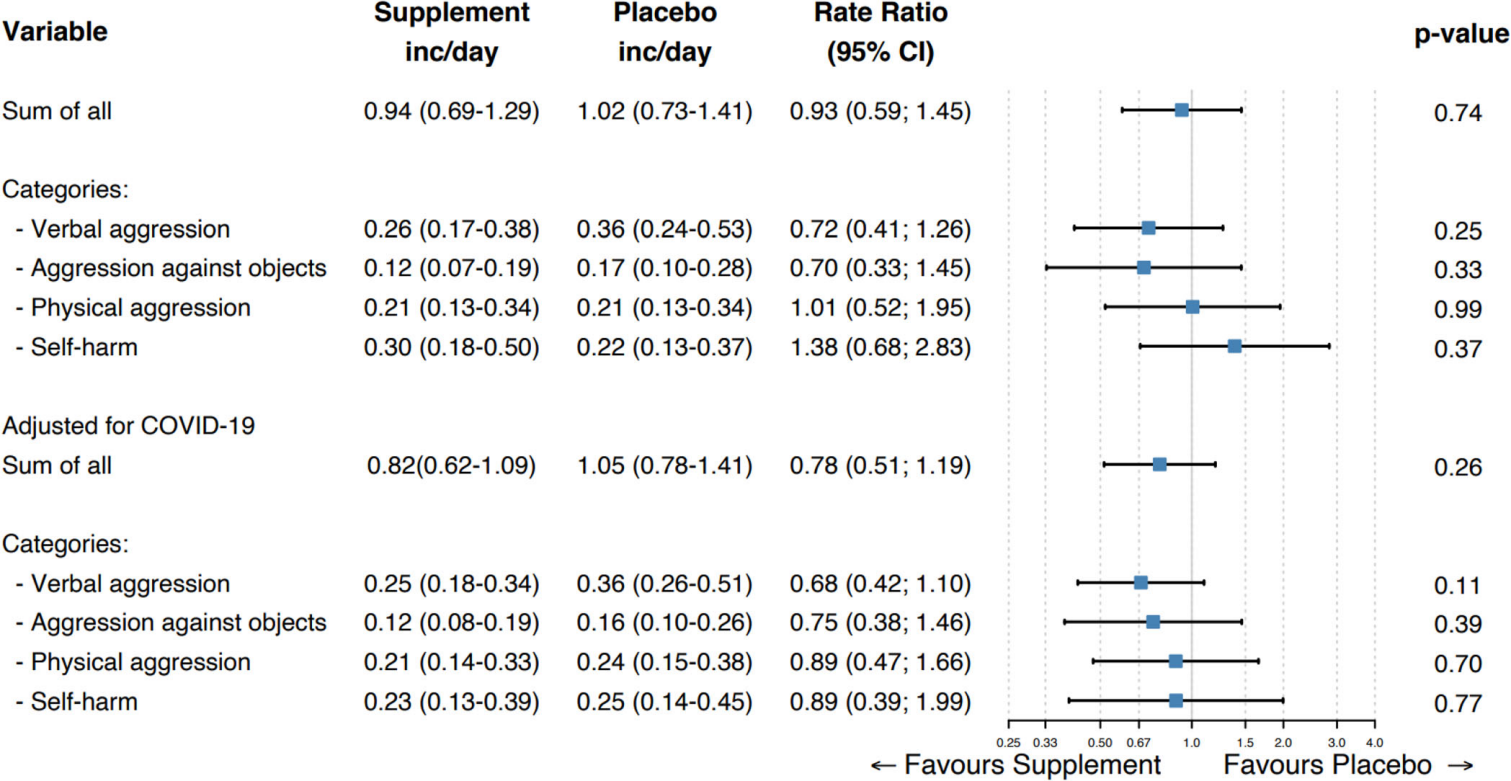
Effects of dietary supplements on aggressive incidents assessed with the MOAS, according to subtype and severity of aggression. The incidents/day are the estimate of the mean in the negative binomial regression analysis [Color figure can be viewed at wileyonlinelibrary.com]

An overview of the effects of supplements on the primary outcome, based on the negative binomial regression analysis, is shown in Figure 2. During the trial period, a total of 13,432 aggressive incidents were registered with the MOAS. There was no significant difference in mean number of incidents per day between those assigned to supplements (0.94; 95% confidence interval [CI]: 0.69–1.29) and those who received placebo (1.02; 95% CI: 0.73–1.41), with a rate ratio of 0.93 (95% CI: 0.59–1.46; *p* = .74). The breakdown by types of aggression (verbal, against objects, physical and self‐harm) did not yield significant differences either.

**FIGURE 3:**
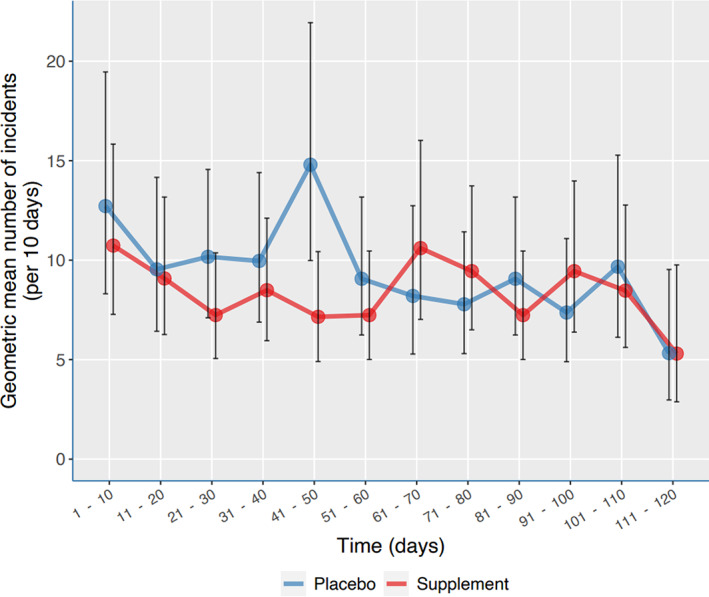
Effects of dietary supplements on aggressive incidents assessed with the MOAS, per period of 10 days. [Color figure can be viewed at wileyonlinelibrary.com]

There was no unambiguous difference in effect between active and placebo group over time, as can be seen in the timeline in Figure 3, which shows the mean number of incidents (with 95% CI's) per 10 days.

### 3.2 Secondary outcomes

The change in QoL over time did not differ significantly between the randomised groups, active arm (mean change = 0.61; 95% CI: −10.48; 11.71) and placebo arm (mean change = 3.0; 95% CI: −8.09; 14.13). For the secondary outcome of changes in aggressive behaviour there was no statistically significant difference between active arm (mean change = −3.58; 95% CI: −14.17; 7.00) and placebo arm (mean change = −2.98; 95% CI: −13.58; 7.62) either.

### 3.3 COVID‐19 outcomes

In total 40 (29.2%) participants were in our trial during COVID‐19 time (57.5% active vs. 42.5% placebo), of which all 24 crossover participants. Adding the COVID‐19 covariate to the models did not change the results significantly IRR 0.78 (95% *CI*: 0.51–1.19; *p* = .28) although there seemed to be a difference in the directions of the effect before and during the pandemic. Pre COVID‐19, the effect was in favour of the active supplements IRR 0.62 (95% CI: .34–1.15), and during the COVID‐19 the effect was in favour of the placebo IRR 1.44 (95% CI: .77–2.70), the interaction ‘treatment’ x ‘COVID‐19’ did not reach statistical significance *B* = −0.84 (95% *CI*: −1.73; 0.06; *p* = .067). Finally, during COVID‐19 there were more aggressive incidents registered then before COVID‐19; IRR 1.99 (95% CI: 1.30; 3.01), especially for physical aggression IRR 2.51 (95% CI: 1.34; 4.70) and self‐harm IRR 3.10 (95% CI: 1.67: 5.76). The samples pre‐ and during COVID‐19 differed significantly on IQ (pre *M* = 50.6 [19.9], during *M* = 33.7 [16.4]; *t* = 4.75, *p* < .01), Age (pre *M* = 22.6 [7.3], during 25.3 [6.6]; *t* = −2.11, *p* = .04), and Diet Quality (pre *M* = 79.8 [16.9], during *M* = 87.6[13.2]; *t* = −2.88, *p* = .01). The difference on baseline BMI did not reach significance.

### 3.4 Adherence

The adherence to the daily intake of the supplements in the total sample was (83.8%), and did not differ significantly between active group (84.4%) and placebo (83.2%).

### 3.5 Blinding

Table 2 shows success of blinding. The vast majority of participants during the 137 treatment trajectories (*n* = 108, 81.2%) and their support staff (*n* = 82, 61.6%) did not guess correctly whether supplements or placebo had been provided. Among the participants and support staff who thought they knew which group they were in, there was no significant difference between the proportion of wrong and correct guesses.

**TABLE 2:** Group assignment x guess of group assignment by participants and support staff

	Participant guess				Support staff guess			
	No idea	Placebo	Active	Total	No idea	Placebo	Active	Total
Active	54 (78.3%)	8 (11.6%)	7 (10.1%)	69	42 (60.9%)	18 (26.1%)	9 (13.0%)	69
Placebo	54 (84.4%)	6 (9.4%)	4 (6.3%)	64	40 (62.5%)	19 (29.7%)	5 (7.8%)	64
Total	108 (81.2%)	14 (10.5%)	11 (8.3%)	133	82 (61.7%)	37 (27.8%)	14 (10.5%)	133

### 3.6 Adverse events

Table 3 shows the number of adverse events reported at the end of the study. The most common symptoms were gastrointestinal problems and lack of energy. The absolute number of participants with at least 1 adverse event in the active group (*n* = 21, 29.6%) did not significantly differ from that in the placebo group (*n* = 21, 31.8%). There were also no significant differences in the total number of adverse events between the two groups.

**TABLE 3:** Number of adverse effects reported at the end upon inquiry

	Active	%	Placebo	%
Gastrointestinal problems	7	10.1	11	17.2
Low energy	7	10.1	4	6.3
Skin‐related problems	3	4.3	2	3.1
Nosebleeds	1	1.4	1	1.6
Headache	3	4.3	1	1.6
Sleeping problems	10	14.5	6	9.4
Participants with any adverse event	21	29.6	21	31.8

## 4 DISCUSSION

In this pragmatic RCT involving people with intellectual disabilities and borderline intellectual functioning, we found no significant difference in the number of aggressive incidents between those assigned to dietary supplements and those assigned to placebo: neither in the total score, nor in the scores broken down by type of aggression. We neither found a significant difference in effectiveness on secondary or safety outcomes. Finally, we found no difference in the total number of adverse reactions reported between the two groups. It should be noted, however, that the number of registered incidents had doubled during the COVID‐19, and we found a trend that the direction of the effect changed during the pandemic, which may have affected our effect estimates.

In the past decades, 11 RCTs have been performed with multivitamin‐mineral supplements as an intervention and aggressive behaviour as an outcome (De Bles et al., 2022; Gesch, 2011; Long & Benton, 2013; Raine et al., 2016; Rucklidge et al., 2018; Schoenthaler et al., 1997; Schoenthaler et al., 2021; Schoenthaler & Bier, 2000; Tammam et al., 2016; Zaalberg et al., 2010). Many different outcome measures have been used to map behaviour, ranging from self‐report (Long & Benton, 2013), and observer report questionnaires (Raine et al., 2016; Rucklidge et al., 2018), to the count of incidents (Schoenthaler et al., 1997; Schoenthaler et al., 2021; Schoenthaler & Bier, 2000), or both (De Bles et al., 2022; Gesch et al., 2002; Tammam et al., 2016; Zaalberg et al., 2010). All but one study (De Bles et al., 2022) had an effect in favour of the supplements on at least one of the outcome measures. The effect may be modified by age. The only study that also included older participants had a null finding (De Bles et al., 2022). The age of the participants in the other studies ranged from 6 to 25 years. A large proportion of the participants in our RCT used psychotropic medication (58.4%). In most previous RCTs people who used psychotropic medication were only a small minority of the sample or were excluded (Raine et al., 2016; Rucklidge et al., 2018; Schoenthaler et al., 1997). An exception was the study by De Bles et al. (2022) in which patients with mental disorders were included. In a post‐hoc subgroup analysis supplements seemed to be less effective in those using antipsychotics (De Bles et al., 2022). We may conclude that all trials differed in multiple ways from each other and from our trial. But trials that excluded the use of psychotropic medication tended to show a larger beneficial effect than the trials that did not.

### 4.1 Strengths and limitations

A strong point of the study was the sample‐wide large number of registered incidents, which protected against floor effects. This was the result of a threshold of a minimum level of aggressive behaviour as an inclusion criterion, and also by weekly monitoring of the daily registrations of incidents. Another strength was the successful blinding, which has been less successful in some previous supplement studies (Long & Benton, 2013; Tammam et al., 2016; Zaalberg et al., 2010).

Some limitations must also be acknowledged. First, a significant portion of our research trajectories (40, 29.2%), including all crossover trials, took place during the COVID pandemic. COVID‐19 and associated restrictions caused major changes in the lives of people with intellectual disabilities, for example, social distancing, closing of the day care centers, and an entry ban for visiting family members (Embregts et al., 2020; Gleason et al., 2021). Behavioural changes as a result of COVID‐19 affected many studies, and may have affected their outcomes (Aman & Pearson, 2020; Stiles‐Shields et al., 2020). In our sample, the number of reported aggression incidents per person during COVID‐19 had doubled and the direction of the effect during COVID‐19 changed direction from in favour of supplement to in favour of the placebo, which was a statistical trend (*p* = .067). The change in effect size was mainly driven by a rise in self‐harm and physical aggression in a subgroup of people with a lower IQ, higher age, and higher diet quality. Explanations for this change of effect direction remains speculative. A second limitation was that only a small and selected sample of participants progressed to the crossover study, with more participants in the active then in the placebo condition. A third limitation is that we do not know much about the washout time of the effect of micronutrients on behaviour, so the participants who took placebo during the crossover may still have benefited from the supplements in the first trial. Finally, we received feedback from the support staff of people with severe to profound intellectual disabilities that they thought the MOAS did not always match with the behaviour of their participants. For example, what is the validity of rating verbal aggression if the participant is not able to speak? Despite the good psychometric properties of the MOAS from previous research, it appears to be difficult to find an instrument that is well suited for measuring aggression of people with severe and those with mild levels of intellectual disabilities.

### 4.2 Conclusions

In this pragmatic trial, we did not find significant differences in the primary and secondary effectiveness between the supplement and placebo arms among people with intellectual disabilities. Since the COVID‐19 pandemic coincided with our trial, we recommend a replication of our study.

## FUNDING INFORMATION

This work was supported by the Healthcare Insurance Fund, The Netherlands (Het Innovatiefonds Zorgverzekeraars) under Grant number 3326. Bonusan donated the trial supplements and placebos. The funders had no further role in the study.

## CONFLICT OF INTEREST

The author declares that there is no conflict of interest.

## PATIENT CONSENT

All participants and/or their legal representatives have given informed consent.

## Supporting information


**Appendix S1:** Supporting Information.

## Data Availability

Data available on request from the author.
