# Supplementary material for: Dietary supplements for aggressive behaviour in people with intellectual disabilities: A randomised controlled crossover trial
Source: J Appl Res Intellect Disabil. 2022 Oct 12;36(1):122–31. doi: 10.1111/jar.13041 (PMC10092216; doi:10.1111/jar.13041)
Supplement: Supplementary file 1 — Appendix S1: Supporting Information. [file JAR-36-122-s001.docx]

**Appendix 1.** Daily dosages of vitamins, minerals and omega-3 fatty acids in RCTs

|  | Schoenthaler 1997 | Schoenthaler 2000 | Gesch 2002 | Zaalberg 2010 | Adams 2011 | Long  2013 | Tammam 2016 | Raine 2016 | Rucklidge 2018 | de Bles 2022 | Gast 2022 |
| --- | --- | --- | --- | --- | --- | --- | --- | --- | --- | --- | --- |
| **Minerals** |  |  |  |  |  |  |  |  |  |  |  |
| **Potassium (mg)** | - | - | 4 | 4 | 50 | 40 | - | 40 | 192 | - | - |
| **Calcium (mg)** | 122 | 200 | 100 | 100 | 100 | 162 | - | 162 | 1056 | - | - |
| **Manganese (mg)** | 3 | 1 | 3 | 3 | 3 | 2 | 2 | 2 | 7.7 | 5 | 0.5 |
| **Iron (mg)** | 18 | 9 | 12 | 12 | - | 5 | 12 | 7.2 | 10.8 | 8 | 4 |
| **Zinc (mg)** | 15 | 8 | 15 | 15 | 12 | 5 | 15 | 8.3 | 38.4 | 7.5 | 10 |
| **Copper (mg)** | 2 | 1 | 2 | 2 | - | 0.5 | 1 | 0.9 | 5.8 | 0.5 | 0.5 |
| **Magnesium (mg)** | 59 | 80 | 30 | 100 | 100 | 100 | 94 | 20 | 480 | - | 70 |
| **Molybdenum (μg)** | 250 | 120 | 250 | 250 | 150 | 50 | - | - | 120 | 13 | 13 |
| **Borium (mg)** | - | - | - | - | - | - | - | - | - | - | 1 |
| **Selenium (μg)** | 100 | 50 | 50 | 50 | 22 | 30 | 55 | - | 168 | 75 | 75 |
| **Chromium (μg)** | 100 | 50 | 200 | 200 | 70 | 40 | 50 | - | 504 | 13 | 100 |
| **Iodine (μg)** | 150 | 75 | 140 | 140 | 100 | 100 | 130 | 160 | 163 | 150 | 80 |
| **Lithium (μg)** | - | - | - | - | 500 | - | - | - | - | - |  |
| **Sulfur (mg)** | - | - | - | - | 500 | - | - | - | - | - |  |
| **Phosphorus (mg)** | - | - | - | - | - | 125 | - | - | 672 | - |  |
| **Chloride (mg)** | - | - | - | - | - | 36.3 | - | - | - | - |  |
|  |  |  |  |  |  |  |  |  |  |  |  |
| **Vitamins** |  |  |  |  |  |  |  |  |  |  |  |
| **A (mg)** | 1.5 | 0.75 | 0.75 | 0.75 | 0.6 | 0.8 | 0.4 | 0.8 | 1.4 | - |  |
| **Βeta carotene mg** | - | – | – | 0.13 | - | - | - | - | - | 6 | 2.9 |
| **B1 (mg)** | 4.5 | 0.75 | 1.2 | 1.2 | 20 | 1.4 | 6 | 1.4 | 48 | 15 | 12 |
| **B2 (mg)** | 5.1 | 0.9 | 1.6 | 1.6 | 20 | 1.75 | 3 | 1.75 | 14.4 | 15 | 16 |
| **B3 (mg)** | 60 | 10 | 18 | 18 | 25 | 20 | 18 | 20 | 72 | 20 | 35 |
| **B5 (mg)** | 30 | 5 | 4 | 4 | 15 | 7.5 | 6 | 7.5 | 24 | 15 | 25 |
| **B6 (mg)** | 30 | 1 | 2 | 2 | 40 | 2 | 8 | 2 | 56 | 5 | 5 |
| **B11 Folic acid (μg)** | 400 | 200 | 400 | 400 | 100 | 200 | 400 | 200 | 640 | 400 | 200 |
| **B12 (μg)** | 18 | 3 | 3 | 3 | 500 | 2.5 | 15 | 2.5 | 720 | 25 | 70 |
| **Biotin (μg)** | - | 150 | 100 | 100 | 150 | 62.5 | 75 | - | 864 | 25 | 25 |

|  | Schoenthaler 1997 | Schoenthaler 2000 | Gesch 2002 | Zaalberg 2010 | Adams 2011 | Long  2013 | Tammam 2016 | Raine 2016 | Rucklidge 2018 | de Bles 2021 | Gast 2022 |
| --- | --- | --- | --- | --- | --- | --- | --- | --- | --- | --- | --- |
| **C (mg)** | 120 | 40 | 60 | 60 | 600 | 100 | 80 | 100 | 480 | 100 | 80 |
| **D3 (μg)** | 5 | 5 | 10 | 5 | 7.5 | 5 | 20 | 5 | 60 | 25 | 15 |
| **E (mg)** | 10 | 10 | 10 | 270 | 15 | - | 8 | 15 | 200 | 53 | 8 |
| **K (mg)** | 50 | - | - | - | - | - | - | - | - | - | 50 |
|  |  |  |  |  |  |  |  |  |  |  |  |
| **Other** |  |  |  |  |  |  |  |  |  |  |  |
| **Inositol (mg)** | 40 | - | - | - | 100 | - | - | - | - | - | - |
| **Benzoic acid (mg)** | 50 | - | - | - | - | - | - | - | - | - | - |
| **Choline (mg)** | 40 | - | - | - | 250 | - | - | - | - | - | - |
| **DMEA (mg)** | - | - | - | - | - | - | - | - | - | - | 10 |
| **Tabashir (mg)** | - | - | - | - | - | - | - | - | - | - | 10 |
| **Chlorella (mg)** | - | - | - | - | - | - | - | - | - | - | 25 |
| **Spirulina (mg)** | - | - | - | - | - | - | - | - | - | - | 25 |
| **MSM (mg)** | - | - | - | - | - | - | - | - | - | - | 15 |
|  |  |  |  |  |  |  |  |  |  |  |  |
| **Fatty acids** |  |  |  |  |  |  |  |  |  |  |  |
| **LA** | - | - | 1260 | - | - | 10 | - | 400 | - | - | - |
| **γ-LA (mg)** | - | - | 160 | 100 | - | - | - | - | - | - | - |
| **EPA (mg)** | - | - | 80 | 400 | - | - | 165 | 200 | - | 307 | 300 |
| **DHA (mg)** | - | - | 44 | 400 | - | 673 | 116 | 300 | - | 175 | 200 |

**Ingredients of the placebo supplements used in the Gast (2022) trial:**

The multivitamin-mineral placebo:

- 13 mg rice bran extract
- 440 mg microcrystalline cellulose
- 0.8 mg riboflavin (to secure the blind)

The omega-3 placebo:

- 500 mg high oleic sunflower oil
- 0.75 mg mixed tocopherols
